# Molecular epidemiology of *Enterocytozoon bieneusi* in humans and domestic animals from Heilongjiang province, China: high animal prevalence, novel genotypes, and possible zoonotic implications in rural households

**DOI:** 10.3389/fcimb.2026.1899714

**Published:** 2026-08-24

**Authors:** Tao Hu, Feiyang Zheng, Weizhe Zhang, Yulu Sun, Hanyun Qin, Liang Zhao, Huazhang Lai, Jingwen Chen, He Li, Bo Yang, Xiaoli Zhang, Fengkun Yang

**Affiliations:** Department of Parasitology, School of Basic Medical Sciences, Harbin Medical University, Harbin, Heilongjiang, China

**Keywords:** China, *Enterocytozoon bieneusi*, genetic diversity, Heilongjiang, prevalence, rural households

## Abstract

**Introduction:**

*Enterocytozoon bieneusi* is a zoonotic parasite causing diarrheal disease in rural settings with frequent human–animal contact.

**Methods:**

We conducted a cross-sectional study in Heilongjiang, China, enrolling 69 households across 10 villages. Humans and domestic animals were sampled. Nested PCR targeting the ITS region was performed. Genotypes were determined by sequencing, and genetic diversity was assessed using DnaSP.

**Results:**

Human prevalence was 11.0% (13/118), while overall animal prevalence was 23.4% (162/693), with sheep being the highest (66.7%), followed by pigs (46.1%) and geese (20.5%). Prevalence was significantly higher in mammals than poultry (32.9% vs. 13.9%; p < 0.001). Sequence analysis yielded 29 distinct genotypes (10 known, 19 novel), with EbpC (36.0%), BEB6 (13.7%), EbpA (13.7%), and D (11.4%) being the most prevalent, most clustering within zoonotic Group 1.

**Discussion:**

Notably, zoonotic genotypes EbpC and EbpA were identified in chickens and geese, extending the known host range of these genotypes. Human–animal genotype sharing was observed in only one household (genotype EbpC between humans, pigs, chickens, and geese), while intraspecies circulation and occasional animal–animal genotype sharing were more common. Phylogenetic and network analyses suggested localized diversification, though single-locus ITS limits definitive evolutionary inference. *E. bieneusi* is highly prevalent in rural domestic animals in Heilongjiang, with genetically diverse and potentially zoonotic genotypes circulating in livestock and poultry. Although household-level human–animal transmission appears limited in this dataset, the presence of shared zoonotic genotypes highlights the need for expanded surveillance incorporating environmental sampling and multi-locus typing to clarify transmission pathways and inform One Health strategies.

## 1 Introduction

Microsporidia are obligate intracellular parasitic microorganisms with a global distribution, and broad host ranges. To date, approximately 200 genera and 1,500 species have been identified (Li et al., 2019a). Among human-pathogenic species, *Enterocytozoon bieneusi* (*E. bieneusi*) predominates, accounting for over 90% of reported human microsporidiosis cases (Taghipour et al., 2022). Infections are most frequently observed in immunocompromised individuals, including AIDS patients, children, and the elderly (Jiang et al., 2023; Zhang et al., 2018; Zhou et al., 2022), but are also detected in immunocompetent populations (Zhou et al., 2022). Beyond human health, *E. bieneusi* infection in livestock causes chronic diarrhea, malnutrition, growth retardation, and reduced productivity, resulting in substantial economic losses to farmers and the agricultural sector. As most of infected hosts often remain asymptomatic, they may shed spores unnoticed, facilitating environmental contamination. Transmission occurs primarily via the fecal–oral route through contaminated water, food, or direct contact with infected animals (Muadica et al., 2020; Naguib et al., 2022; Prasertbun et al., 2017; Salamandane et al., 2023; Wang et al., 2024), with clinical manifestations ranging from asymptomatic carriage to severe diarrhea, particularly in vulnerable hosts (Liu et al., 2017).

Molecular characterization of *E. bieneusi* relies on PCR-based genotyping of the highly polymorphic internal transcribed spacer (ITS) region of the small subunit ribosomal RNA (SSU rRNA) gene, from which over 500 genotypes have been identified to date (Udonsom et al., 2019). Phylogenetic analysis classifies these genotypes into 15 distinct groups (Groups 1–15) based on isolates from humans, livestock, companion animals, wild mammals, birds, and water sources (Jiang et al., 2024). Groups 1 and 2 encompass most zoonotic genotypes with broad host ranges, while Groups 3–15 exhibit stronger host specificity and limited or unknown zoonotic potential (Li et al., 2019a; Li and Xiao, 2019; Jiang et al., 2024). Globally, more than 819 genotypes have been documented across 210 animal species in over 50 countries, with at least 184 genotypes reported in humans from 31 countries, among these, 58 genotypes are considered zoonotic, posing potential risks to both human and animal health (Koehler et al., 2022). Major zoonotic genotypes include D, EbpC, EbpA, BEB6, I, and J, with D, A, and Type IV most frequently associated with human infections (Koehler et al., 2022).

Given the widespread distribution of *E. bieneusi* in livestock, occupational exposure represents a significant risk for zoonotic transmission. Farmers, veterinarians, slaughterhouse workers, and other individuals with frequent direct contact with domestic animals are at increased risk of infection. Previous studies have demonstrated that close contact with pigs or pig-farm-contaminated environments significantly elevates the risk of microsporidial infection, particularly in immunocompromised populations (Li et al., 2021a, Li et al., 2025). Therefore, enhanced hygiene protection measures, regular health surveillance for high-risk occupational groups, and improved fecal waste management on farms are essential to reduce the risk of zoonotic transmission. These preventive strategies are particularly important in rural areas where mixed livestock farming and close human–animal contact are common practices (Li and Xiao, 2020; Ye et al., 2025).

In China, 18 molecular epidemiological studies across 11 provinces have reported human prevalence ranging from 0.23% to 24.0% in immunocompetent individuals, with genotypes D and EbpC widely distributed (Ding et al., 2018; Gong et al., 2019; Li et al., 2021a; Liu et al., 2014, Liu et al., 2017; Qiu et al., 2017; Qi et al., 2020; Wang et al., 2013a, Wang et al., 2013b, Wang et al., 2017; Yang et al., 2014; Yan et al., 2021; Yu et al., 2019; Zang et al., 2021; Zhang et al., 2017; Zhang et al., 2011; Zhao et al., 2022; Zhou et al., 2022) (**Supplementary Table 1**). Meanwhile, animal infections have been reported in 18 provinces, four autonomous regions, and three municipalities (Li et al., 2025; Qiu et al., 2019). Domestic animals serve as important reservoirs for *E. bieneusi*. Pigs show particularly high prevalence (up to 89.5% in Heilongjiang) and harbor predominantly Group 1 genotypes (EbpC, EbpA, D, G, H, O, Henan-IV, CS-4) that also occur in humans (Li et al., 2020; Zhao et al., 2014). Ruminants carry diverse genotypes, mainly Group 2 (BEB4, BEB6, I, J), which are also detected in humans (Li et al., 2019, Li et al., 2019b; Zhang et al., 2011). Although poultry generally show lower prevalence, birds can carry Group 1 genotypes with zoonotic potential (Li et al., 2019; Ruan et al., 2021). In neighboring Jilin province, a recent molecular epidemiological survey of pigs revealed an overall *E. bieneusi* prevalence of 5.97% (62/1039), with 12 genotypes identified, including EbpA, EbpC, D, H, and G documented in humans, all of which belong to Group 1 (Kan, 2023). In sika deer from the Changchun region of Jilin province, genotypes BEB6, EbpC, and J have been detected, with BEB6 as the predominant genotype (Li et al., 2021b). Furthermore, studies conducted across Northeast China (Heilongjiang, Jilin, and Inner Mongolia) have reported that pigs carry EbpC, D, and Henan IV—known zoonotic genotypes—at high prevalence (Li et al., 2014; Wan et al., 2016).

In the present study, the major zoonotic genotypes identified were also D, EbpC, and EbpA, all of which were detected in both human and domestic animal samples. Among these, EbpC was the most prevalent genotype in humans, pigs, cattle, dogs, chickens and geese. However, host−specific genotypes varied considerably: pigs harbored the highest diversity with 12 genotypes, predominant EbpC and EbpA; sheep carried three genotypes, with BEB6 being the dominant one; and poultry also showed substantial diversity, with chickens and geese harboring nine and six genotypes, respectively, in which EbpC and EbpA were the predominant ones. These findings indicate that possible zoonotic genotypes are widely distributed in livestock and poultry in Heilongjiang province and neighboring provinces, underscoring for potential cross-species transmission and the importance of surveillance at the human–animal interface.

Identical genotypes in humans and cohabiting animals suggest potential household-level zoonotic transmission (Cama et al., 2007). However, despite numerous regional surveys, studies that simultaneously characterize *E. bieneusi* in cohabiting humans and domestic animals in Heilongjiang remain limited. The extent of genotype sharing between humans and animals at the household level, and the potential role of domestic animals as sources of human infection, require further investigation. Therefore, we conducted a cross-sectional study in rural households in Heilongjiang: (i) estimate the prevalence of *E. bieneusi* in humans and domestic animals; (ii) characterize genotype diversity and document genotype sharing between humans and animals, as well as among different animal species; and (iii) assess zoonotic risks.

## 2 Methods

### 2.1 Study regions in Heilongjiang province, China

The study was conducted in Heilongjiang, a province in northeastern China. It has a temperate monsoon climate (annual average temperature: 1–2 °C) with long, harsh winters and short summers. Heilongjiang is a key agricultural and livestock production area, where pigs and chickens are the most common domestic animals. Pigs and cattle are mainly raised in confinement, while poultry (chickens, ducks, and geese) are typically free-ranging. Centralized wastewater treatment remains limited in remote areas.

### 2.2 Study design and sample collection

**Figure 1:**
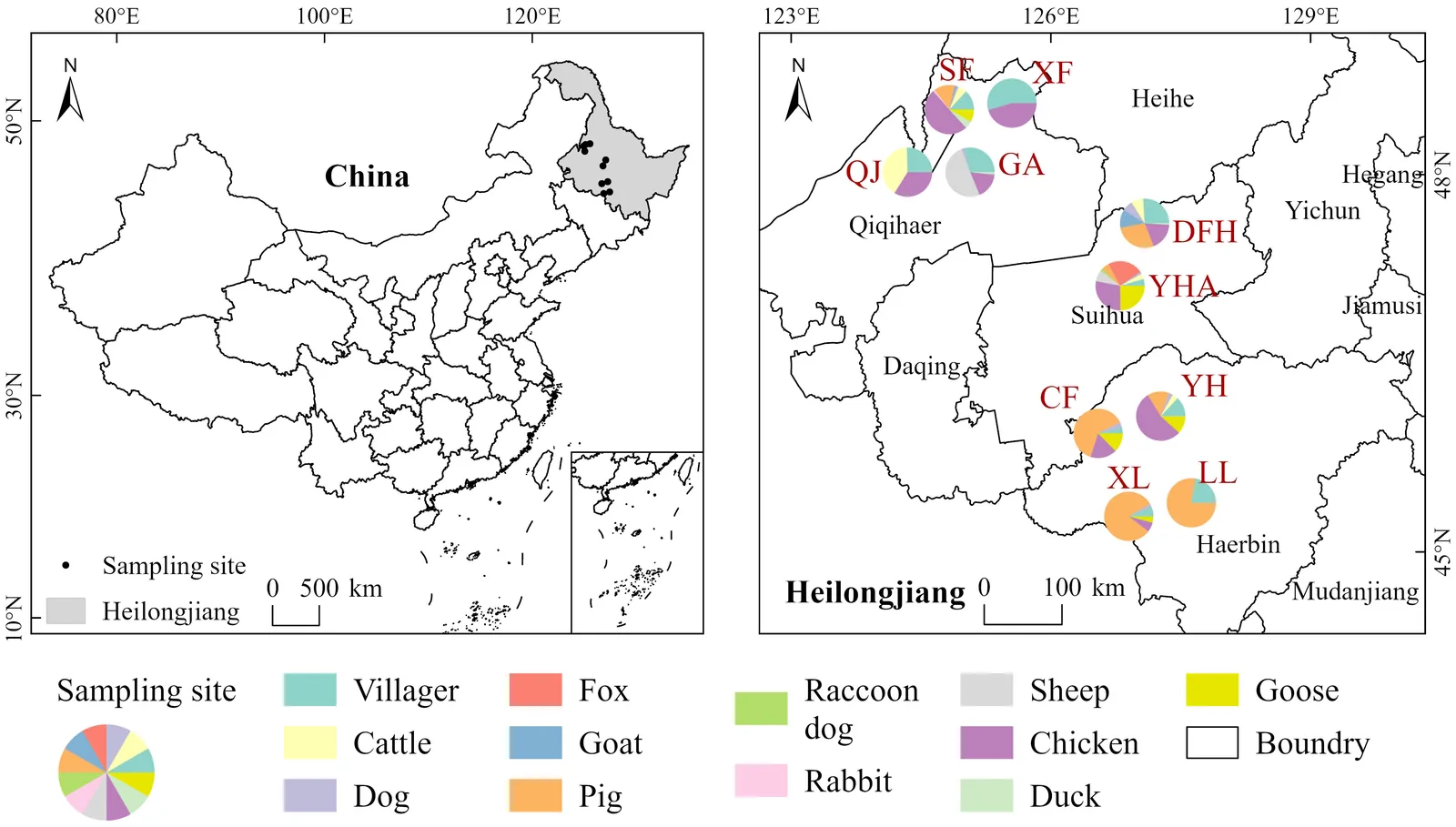
Geographic illustration of the sample collection sites in this study. The map shows the 10 villages in Heilongjiang, China, where human and animal fecal samples were collected. *Abbreviations*: Harbin city: CF, Changfa village; YH, Yuhe village; LL, Lalin village; XL, Xinglong village. Suihua city: DFH, Dongfanghong village; YHA, Youhao village. Qiqihar city: GA, Gongan village; QJ, Qianjin village; SF, Shuangfa village; XF, Xianfeng village.

A cross-sectional survey, stratified cluster study was conducted from November 2020 to November 2024 across 10 villages in Heilongjiang, China (**Figure 1**). Village and household selection was primarily convenience-based, a pragmatic approach necessary to facilitate community participation in this rural field setting. Sampling was conducted in rural areas in the cities of Harbin, Qiqihar, and Suihua. Among these 69 households, both humans and animals were sampled in 44; samples were collected exclusively from animals in 5 and exclusively from humans in 20.

Human sampling: All consenting permanent residents (those who had resided in the household for ≥6 months) were included. A total of 118 human samples were collected. Animal sampling: Species-specific sampling caps were applied based on preliminary field observations of average herd/flock sizes. For large-population species per household (pigs, chickens, sheep, and foxes), approximately one-third of the animals were randomly selected. For small-population species (cattle, goats, dogs, rabbits, raccoon dogs, ducks, and geese), all animals present were sampled. A total of 693 animal samples were collected, comprising 347 mammalian samples (pigs, cattle, goats, sheep, dogs, foxes, raccoon dogs, and rabbits) and 346 poultry samples (chickens, ducks, and geese) (**Table 1**).

**Table 1:** Prevalence and genotype distribution of *E. bieneusi* in humans and domestic animals, by host species.

Hosts		Examined no.	No. of positive (%)	Genotype (n)	
				Known	Novel
Humans		118	13 (11.0)	EbpC, D, EbpA	CHLJ-H1-H5
Mammals	Pigs	167	77 (46.1)	EbpC, D, EbpA, H, PigEBITS5, Henan-III	CHLJ-H4, CHLJP1-P5
	Cattle	51	3 (5.9)	EbpC	CHLJ-H1
	Sheep	45	30 (66.7)	BEB6, CHS7	CHLJ-S1
	Goats	14	2 (14.3)	COS-II	
	Foxes	46	1 (2.2)	D	
	Dogs	19	1 (5.3)	EbpC	
	Rabbits	2	0 (0.0)		
	Raccoon dogs	3	0 (0.0)		
	Subtotal	347	114 (32.9)		
Birds	Chickens	253	31 (12.3)	EbpC*, EbpA*, H*, CHN-H1*, D	CHLJ-C1-C4
	Ducks	10	0 (0.0)		
	Geese	83	17 (20.5)	EbpA*, EbpC*	CHLJ-E1-E4
	Subtotal	346	48 (13.9)		
Total		693	162 (23.4)	BEB6, CHN-H1, COS-II, CHS7, D, EbpA, EbpC, H, Henan-III, PigEBITS5	CHLJ-C1-C4, CHLJ-E1-E4, CHLJ-H1-H5, CHLJ-S1, CHLJP1-P5

HLJ, Heilongjiang Province; CHLJ, novel genotypes from Heilongjiang; H, human; C, chicken; E, goose; P, pig; Sh, sheep; S, goat. *Indicates genotypes detected for the first time in this host.

None of the participants or animals exhibited clinical signs of diarrhea at the time of sampling. Each fecal sample was collected carefully from the upper portion of fresh feces deposited on the ground after defecation by using disposable gloves to avoid contamination or other environmental sources. Immediately after collection, samples were kept individually in a sterile container, on which the name of the human or animal type, collection date, and village of origin were recorded.

### 2.3 Livestock management and sample handling

In Heilongjiang, dogs, chickens, geese, and ducks were primarily free-ranging, while pigs, cattle, goats, foxes, and rabbits were raised in confinement. All samples were placed in ice-packed insulated containers, transported to the laboratory, and stored at 4 °C until DNA extraction.

### 2.4 DNA extraction and PCR amplification

Approximately 200 mg of each fecal sample was washed three times with distilled water by means of centrifugation at 1,500× g for 10 min at room temperature. Genomic DNA was extracted using the QIAamp DNA Stool Mini Kit (Qiagen, Hilden, Germany), according to the manufacturer’s instructions, and extracted DNA was stored at -20 °C. *E. bieneusi* was detected by nested PCR targeting an approximately 392-bp fragment containing the complete ITS and partial sequences of the small and large subunits rRNA genes, using primers previously described (Mirjalali et al., 2015; Sulaiman et al., 2003). Each PCR was conducted in duplicate with nuclease-free water as a negative control and human-derived *E. bieneusi* isolate (genotype: D) as a positive control. PCR products were electrophoresed on 1.5% agarose gels, stained with Gel Stain (TransGen Biotech, China), and visualized under UV light.

### 2.5 Sequence analyses

All products positive by secondary PCR were bidirectionally sequenced using the secondary PCR primers on an ABI PRISM 3730 DNA Analyzer (Applied Biosystems, Waltham, MA, USA), with the BigDye Terminator version 3.1 Cycle Sequencing Kit (Applied Biosystems).

Sequences were analyzed using ChromasPro version 1.62 (Technelysium, Australia) and compared with reference sequences through BLAST (NCBI). Multiple sequence alignments were performed using ClustalW against representative *E. bieneusi* genotypes from GenBank to determine genotype identity. Sequences with ambiguous double peaks, low signal-to-noise ratios, or unclear base calls were re-amplified and re-sequenced from the original DNA template. Samples that remained ambiguous after two repeat sequencing attempts were excluded from further analysis. Only sequences with clear single peaks and a Phred quality score ≥ 20 were retained for genotype assignment.

### 2.6 Nucleotide sequence homology, genetic diversity and possible recombination analyses

Sequences were edited and aligned using ChromasPro v1.62 and MEGA 6.0. Novel genotypes were defined as sequences differing by ≥ 1 consistent single nucleotide polymorphism (SNP) across bidirectional reads compared to known GenBank genotypes (Li and Xiao, 2019; Santín and Fayer, 2011). Genetic diversity parameters, including haplotype diversity (Hd) and the average number of nucleotide differences (k), were calculated using DnaSP version 6.12.03 (Rozas et al., 2017). Recombination analysis was performed using the four-gamete test (Hudson and Kaplan, 1985) implemented in DnaSP to estimate the minimum number of potential recombination events (Rm). This test identifies pairs of polymorphic sites exhibiting all four gametic combinations (00, 01, 10, 11), which are incompatible with a mutation-only model. The four-gamete test on short ITS sequences can indicate incompatibility among polymorphic sites, but it does not by itself demonstrate biologically frequent recombination.

### 2.7 Phylogenetic analysis and haplotype network analyses

Phylogenetic analyses were performed using MEGA software (version 12). Prior to tree construction, the optimal nucleotide substitution model was determined using the “Find Best DNA/Protein Models (ML)” function. The Bayesian Information Criterion (BIC) and the corrected Akaike Information Criterion (AICc) were calculated for 24 candidate nucleotide substitution models. The model with the lowest BIC score was selected as the best-fit model. Based on these criteria, the Tamura-Nei 93 model with a discrete Gamma distribution (+G) and a proportion of invariable sites (+I) [TN93+G+I] was identified as the optimal model for our dataset. Maximum Likelihood (ML) phylogenetic trees were constructed using the TN93+G+I model with 1,000 bootstrap replicates to assess nodal support. Haplotype relationships were further explored with a Median**-**joining network generated in NETWORK version 10.0.0 (Bandelt et al., 1999).

### 2.8 Statistical analysis

Study data were analyzed using IBM SPSS Statistics version 25 (IBM Corporation, Armonk, NY, USA). In the present study, sparse data caused expected cell counts in contingency tables to be < 5. Fisher’s exact test was used to determine the association between *E. bieneusi* infection and each categorical variable (gender, age, seasons, eating unwashed vegetables and fruits, and contact with animals). All prevalence was calculated with 95% CIs using the Wilson score method. Meanwhile, differences in prevalence between groups (e.g., mammals vs. poultry) were assessed using the χ^2^ test with two-sided P values. 95% confidence intervals were calculated. *p* < 0.05 was considered statistically significant.

## 3 Results

### 3.1 Prevalence of *E. bieneusi*

*E. bieneusi* was detected in human, six mammalian hosts (pigs, cattle, sheep, goats, dogs, foxes) and two avian hosts (chickens, geese). Overall prevalence was 21.6% (175/811), comprising 11.0% (13/118) in humans and 23.4% (162/693) in domestic animals. Among mammals, prevalence was 32.9% (114/347), with the highest rate in sheep (66.7%, 30/45), followed by pigs (46.1%, 77/167), goats (14.3%, 2/14), cattle (5.9%, 3/51), dogs (5.3%, 1/19), and foxes (2.2%, 1/46). Among poultry, prevalence was 13.9% (48/346), with geese (20.5%, 17/83) showing higher rates than chickens (12.3%, 31/253). No positive samples were detected in rabbits, raccoon dogs, or ducks (**Table 1**).

Prevalence was significantly higher in mammals than in poultry (32.9% vs. 13.9%; χ^2^=34.8, CI: 0.128–0.251; *p* < 0.001). By contrast, no significant associations were observed between *E. bieneusi* prevalence in humans and any of the examined variables (gender, age, season, eating unwashed vegetables and fruits, or contact with animals), with all *p* values >0.05. Although prevalence was numerically higher among females (13.7%), individuals aged 45–69 years (12.0%), during winter (12.5%), in those reporting consumption of unwashed produce (12.3%), and among those with close animal contact (12.5%), none of these differences reached statistical significance (**Table 2**).

**Table 2:** Demographic and exposure characteristics of participants by *E. bieneusi* infection status.

Variable	Total (N = 118)	Positive (N = 13)	95% CI	Negative (N = 105)	P-value
Gender, n (%)					0.555
Male	67	6 (9.0)	3.6–18.8	61 (91.0)	
Female	51	7 (13.7)	6.0–26.8	44 (86.3)	
Age group, n (%)					0.684
<45	18	1 (5.6)	0.3–28.0	17 (94.4)	
45-69	83	10 (12.1)	6.2–21.4	73 (87.9)	
>69	17	2 (11.8)	2.1–36.9	15 (88.2)	
Season, n (%)					0.545
Winter	80	10 (12.5)	6.5–22.2	70 (87.5)	
Summer	38	3 (7.9)	2.1–21.8	35 (92.1)	
Unwashed vegetables/fruits, n (%)					0.770
Yes	65	8 (12.3)	5.9–23.1	57 (87.7)	
No	53	5 (9.4)	3.7–21.1	48 (90.6)	
Animal contact, n (%)					0.282
Yes	96	12 (12.5)	6.8–21.3	84 (87.5)	
No	22	1 (4.5)	0.2–23.4	21 (95.5)	

### 3.2 Genotype identification, host distribution, and genetic diversity analysis

Sequence analysis of 175 positive specimens yielded 29 distinct ITS genotypes (29 haplotypes), comprising 10 known and 19 novel genotypes (**Supplementary Table 2**). The most prevalent genotypes were EbpC (63/175, 36.0%), BEB6 (24/175, 13.7%), and EbpA (24/175, 13.7%), followed by D (20/175, 11.4%), and H (6/175, 3.4%), with the remaining known genotypes each accounting for < 3% of positive samples. Novel genotypes were predominantly single-nucleotide variants of known zoonotic genotypes (EbpC, EbpA, D, Henan I, JLD XII, BLC4, and H), differing by 1–4 SNPs across the ITS region. Host-specific distribution patterns were observed (**Supplementary Table 2**).

In humans, eight genotypes were detected, including three known zoonotic genotypes (EbpC, EbpA, D) and five novel genotypes (CHLJ-H1-H5). In animals, pigs harbored the greatest genotype diversity (12 genotypes), followed by chickens (9), geese (6), sheep (3), cattle (2). Single genotypes were detected in goats (COS-II), dogs (EbpC), and foxes (D). Notably, we identified genotypes CHN-H1, EbpC, EbpA, and H in chickens, and EbpA and EbpC in geese—host species for which these genotypes (EbpC, EbpA, H, CHN-H1) have rarely or never been reported, thereby expanding the known host range of this pathogen (**Table 1**).

Using DnaSP, the haplotype diversity (Hd) was 0.988 and the average number of nucleotide differences (k) was 9.345, indicating a high level of genetic diversity. Among the 29 ITS sequences analyzed, a total of 49 polymorphic sites were identified. The four-gamete test detected incompatibility among these sites, yielding a minimum recombination event estimate of Rm = 5. This finding is suggestive of potential recombination events within the studied population. Accordingly, these findings should be considered preliminary and hypothesis-generating, and should be validated using multi-locus sequence typing approaches.

### 3.3 Limited cross-species genotype overlap and predominantly intraspecies genotype distribution of *E. bieneusi*

Of the 69 households surveyed, 33 (47.8%) had at least one *E. bieneusi*-positive human or animal. Identical genotypes shared between humans and cohabiting animals were observed in only one household (CF, Household 1), where zoonotic genotype EbpC was detected in two villagers and multiple animals (19 pigs, 11 chickens, 7 geese), suggesting possible genotype overlap or shared exposure. Although direct evidence for inter-species (human-animals) transmission at the household level is limited (observed in only one household), the predominance of shared Group 1 genotypes in both humans and animals highlights the need for further studies incorporating environmental sampling and multi-locus typing to clarify transmission dynamics. Notably, genotype H was shared by chickens and pigs also found in household CF/1. Additionally, cross-host sharing was also observed in two other households: genotype D between geese and pigs (YHA/5), and genotype EbpC between dogs and pigs (XL/2). Although human–animal genotype overlap occurred in only one of 44 households, the occurrence of three independent cross-species sharing events across different animal combinations supports the occurrence of inter-species transmission at the household level. Evidence consistent with intraspecific genotype sharing was noted in eight households, where identical genotypes recurred within the same species—D, H, PigEBITS5, EbpA, EbpC, and CHLJ**-**P1 in pigs; BEB6 in sheep; and EbpC in geese—indicating active circulation within animal populations. Detailed household-level genotype distributions are provided in **Table 3**.

**Table 3:** Household-level distribution of *E. bieneusi* genotypes in humans and domestic animals across five villages with positive samples.

Provinces	Village/Family ID	*E. bieneusi* genotypes	
		Human (n)	Animal (n)
Heilongjiang	CF/1	**EbpC**: H111, H112 (2)	**EbpC**: Goose (7); Chicken (11); Pig (19); **H**: Chicken (1); Pig (5); **CHLJ-C4**: Chicken (1); **CHLJ-P5**: Pig (1); **PigEBITS5**: Pig (1); **CHLJ-H4**: Pig(1); **EbpA**: Pig (9); **CHLJ-P1**: Pig (2)
	LL/1	0	**CHLJ-P1**: Pig (1); **EbpC**: Pig (1); **EbpA**: Pig (1)
	YHA/1	0	**EbpC**: Chicken (2); **CHLJ-E4**: Goose (1)
	YHA/5	0	**EbpA**: Chicken (2); **D**: Goose (1), Pig (3)
	YHA/6	0	**D**: Chicken (2); **EbpA**: Chicken (2); **CHLJ-C1**: Chicken (1); **EbpC**: Goose (1); **CHLJ-E2**: Goose (1)
	YHA/7	0	**BEB6:** Sheep (2)
	XL/1	0	**CHLJ-P2**: Pigs (1); **PigEBITS5**: Pig (2); **EbpC**: Pig (2); **CHLJ-P3**: Pig (1)
	XL/2	**CHLJ-H5**: H116 (1)	**EbpC**: Dog (1); Goose (2); Pig (6); **EbpA**: Pig (4); **CHLJ-P4**: Pig (1); **Henan-III**: Pig (1)
	QJ/3	0	**EbpC**: Cattle (1)
	QJ/4	**EbpC**: H65 (1)	0
	QJ/5	**EbpC**: H68 (1)	0
	QJ/8	0	**EbpC**: Chicken (2); **CHLJ-C3**: Chicken (1)

H, human; B, cattle; C, chicken; E, goose; P, pig; Sh, sheep; S, goat. Bold values indicate the genotypes identified in this study.

### 3.4 Phylogenetic clustering, and median-joining network analyses of *E. bieneusi* ITS genotypes

**Figure 2:**
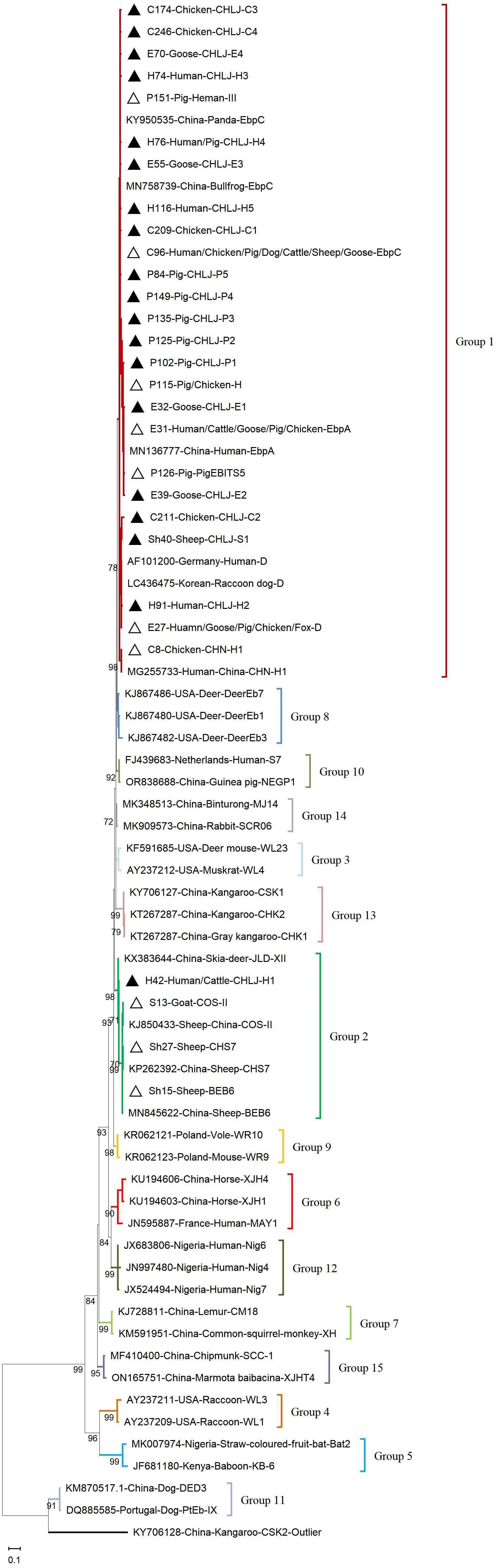
Phylogenetic relationships of *E. bieneusi* genotypes based on ITS gene sequences. A Maximum Likelihood (ML) tree was constructed using the TN93+G+I model, selected as the best-fit model based on the lowest BIC score; Bootstrap values >70% (1,000 replicates) are shown at branch nodes. The *E. bieneusi* genotype CSK2 (accession no. KY706128) from Chinese kangaroo was used as the outgroup. Reference sequences are annotated with their GenBank accession numbers, country, and host. Novel genotypes identified in this study are indicated by solid black triangles; known genotypes are indicated by hollow black triangles. Different genetic groups (Groups 1–15) are distinguished by colored symbols. The scale bar represents nucleotide substitutions per site.

Maximum Likelihood analysis of ITS sequences grouped the 29 genotypes into two clades (**Figure 2**). Phylogenetic analysis placed the novel poultry genotype CHLJ-C1–4 within the Group 1 clade. Given the limited reports of *E. bieneusi* in poultry and the fact that poultry are not considered a typical host for this parasite, we tentatively designated this genotype as Group 1-like. Group 1 (zoonotic) comprised 79.4% (139/175) of isolates, including 7 known and 18 novel genotypes. Group 2 (ruminant-associated) accounted for 20.6% (36/175), including known genotypes (BEB6, CHS7, COS-II), and novel CHLJ-H1. At the household level, clustering of several genotype sequences from humans and animals supports probable intra- and interspecies transmission. In household GA4, sequences of the human-derived CHLJ**-**H2, the sheep-derived CHLJ**-**S1, and D clustered together with strong support; in XL2, human-derived CHLJ**-**H5, pig-derived CHLJ**-**P4 and Henan-III, and EbpC from dog, goose, and pig isolates formed a single well-supported branch. These patterns are consistent with potential cross-species transmission within affected households.

**Figure 3:**
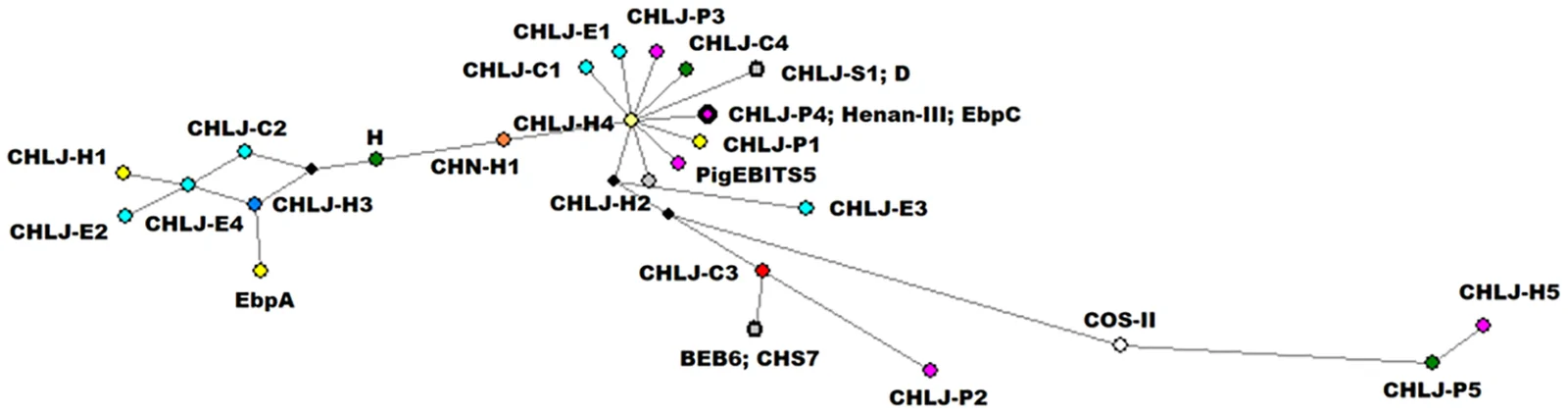
Median-joining network analysis of 29 ITS gene sequences of *E. bieneusi*. Each circle represents a unique genotype, and the thickness of the circle border is proportional to the number of isolates. Circle colors indicate the source village of the isolates. Yellow circles represent genotypes that were present in multiple villages without a clear geographic pattern, whereas each other color represents a different village of origin: e.g., white for SF, blue for YHA, green for CF, gray for GA, purple for XL, orange for YH, dark blue for XF, red for QJ.

Median-joining network analysis of the 29 ITS gene sequences revealed a star-like topology centered on the genotype CHLJ**-**H4 from one pig and one human (**Figure 3**). From this central node, numerous closely related genotypes radiated outward, forming a typical hub-and-spoke structure. The topological positions of these haplotypes are fully consistent with their SNP differences from the central haplotype: haplotypes with fewer SNP differences are positioned closer to the central haplotype CHLJ-H4, while those with more SNP differences are located at greater network distances. This pattern is consistent with localized diversification from a central sequence, though this interpretation remains preliminary given the ITS fragment length (~243 bp). Most genotypes were village-specific, suggesting predominantly local diversification, while only a few (CHLJ-H1, CHLJ-P1, EbpA, EbpC, BEB6, and D) were found across multiple villages, implying limited inter-village transmission based on current data. Higher-resolution approaches are required to confirm these findings.

## 4 Discussion

### 4.1 Domestic animals and poultry as potential reservoirs

In the present study, the prevalence of *E. bieneusi* among the human population in Heilongjiang Province was 11.0%. This rate falls within the global range reported for immunocompetent populations (1.4%–46.7%) (Zhang et al., 2021) and is comparable to reports from rural settings in Chinese (e.g., 8.3% in Yunnan) (Gong et al., 2019), but lower than those in high-risk occupational groups such as animal facility workers (24.0%) (Li et al., 2021a). Most participants lived in households with livestock and poultry, where close contact may facilitate exposure (Li et al., 2019, Li et al., 2020). This study reveals a markedly higher prevalence of *E. bieneusi* in mammals than in poultry, underscoring the importance of mammalian hosts as primary reservoirs in the study area. Conversely, none of the examined individual-level risk factors—including animal contact—were significantly associated with infection. The small number of positive isolates (n=13, 11.0%) limited statistical power and precluded multivariable regression analyses. Therefore, the relative contributions of behavioral exposures cannot be disentangled from the current data. We therefore propose that future surveillance efforts incorporate environmental sampling alongside host screening, and that larger-scale studies be conducted to further disentangle the relative contributions of behavioral versus environmental exposures.

Domestic animals in Heilongjiang exhibited substantial variation in prevalence, with mammals (32.9%) showing significantly higher rates than poultry (13.9%). Sheep had the highest prevalence (66.7%), consistent with previous studies. Sheep are susceptible to *E. bieneusi* (Li et al., 2019; Ye et al., 2015). Pigs showed the second highest infection rate (46.1%), consistent with their recognized role as major reservoirs globally (Li, DF 2019; Li et al., 2014; Wan et al., 2016; Wang et al., 2019; Zhou et al., 2020). Goats (14.3%) and geese (20.5%) also showed notable infection rates, while foxes (2.2%) had the lowest. Poultry in this study showed an overall prevalence of 13.9%, with geese (20.5%) exhibiting higher rates than chickens (12.3%). These findings align with reported ranges in China (chickens: 1.94%–14.3%; geese: 13.9%–30.8%) (Cao et al., 2020; Reetz et al., 2002; Zhao et al., 2016, Zhao et al., 2019) and suggest that waterfowl may contribute to transmission pathways. Notably, no infection was detected in rabbits, raccoon dogs, or ducks in this study. However, several factors may contribute to these negative findings. First, the sample sizes for these host species were relatively small, which may limit the power to detect infections at low prevalence levels. Second, species-specific susceptibility may contribute to the negative results in ducks. To our knowledge, no previous studies have reported *E. bieneusi* infection in ducks, suggesting that ducks may be less susceptible or resistant to this pathogen compared with other poultry species such as geese and chickens. Therefore, these negative results should be interpreted with caution, and further studies with larger sample sizes and longitudinal sampling are warranted to better assess the infection status of these species.

### 4.2 Genotype diversity and zoonotic implications

ITS sequence analysis revealed substantial genetic diversity within the study population (Hd = 0.988; k = 9.345). Application of the four-gamete test detected incompatibility among polymorphic sites (Rm = 5), raising the possibility of recombination event. However, because ITS is a short, single-locus marker (~243 bp), such analyses cannot definitively distinguish recombination from other evolutionary processes (e.g., mutation or convergent evolution). Therefore, while our data are suggestive of genetic diversification, confirmation of recombination and its contribution to host adaptation requires higher-resolution typing methods. A total of 19 novel genotypes were identified, most of which cluster within zoonotic Group 1, thereby extending the known genetic diversity of *E. bieneusi* in Heilongjiang Province, China. The majority of these novel variants differ from known zoonotic genotypes by only 1–4 SNPs, indicating ongoing predominantly localized diversification under mixed host and environment pressures that may enhance zoonotic adaptation (Li and Xiao, 2019). Additionally, we detected several genotypes in poultry—CHN-H1, EbpC, EbpA, and H in chickens, and EbpA and EbpC in geese—that have been predominantly reported in mammalian hosts (Cao et al., 2020; da Cunha et al., 2016; Ercan et al., 2020; Mohammad Rahimi et al., 2021; Reetz et al., 2002; Zhao et al., 2016, Zhao et al., 2019). These findings extend the known host associations of these genotypes and point to potential transmission plasticity between mammals and birds.

Median-joining network analysis revealed a star-like topology centered on CHLJ-H4, with satellite genotypes differing by one or a few mutational steps. This topology is suggestive of CHLJ-H4 serving as a putative ancestral genotype, from which local variants have diversified—potentially through mutation accumulation—within individual villages or households. Notably, most novel genotypes were village-specific, while only a few (e.g., EbpA, EbpC, BEB6, and D) were found across multiple villages without clear geographic structuring. This pattern raises the possibility of predominantly localized diversification, with limited evidence for widespread inter-village transmission based on the current ITS data.

However, we emphasize that these interpretations are preliminary. The ITS locus is short (~243 bp) and cannot distinguish between mutation, recombination, and convergent evolution. Given that *E. bieneusi* is known to undergo recombination, high-resolution approaches such as MLST are essential to validate the apparent star-like structure, confirm the putative ancestral status of CHLJ-H4, and resolve the respective roles of mutation versus potential recombination in shaping local genetic diversity.

### 4.3 Study limitations

This study has several limitations that should be considered when interpreting the findings. First, the cross-sectional design precludes inference of transmission directionality; second, only one household showed identical genotype sharing between humans and animals, which constrains our ability to draw conclusions about household-level transmission networks; third, the absence of environmental samples (water, soil) prevents comprehensive assessment of transmission pathways. Finally, the phylogenetic analyses in this study were based on a single-locus ITS marker (~243 bp), which has inherent limitations for evolutionary inference. *E. bieneusi* is known to exhibit recombination, and short sequence fragments cannot reliably distinguish true clonal lineages from recombination or convergent evolution. Therefore, the ITS tree presented here serves as an exploratory tool for visualizing genotype similarity, rather than a definitive reconstruction of phylogenetic history.

### 4.4 Future directions

To validate these preliminary findings, future studies should prioritize three key areas: first, implementing longitudinal, multi-locus sequence typing (MLST)-based surveillance across expanded geographic and host ranges to resolve transmission dynamics and potential recombination events; second, integrating environmental sampling (water and soil) to clarify environmental transmission routes; and third, broadening host coverage to better assess cross-species spillover risks. Collectively, these large-scale, multi-disciplinary efforts are essential to confirm localized diversification patterns and to underpin evidence-based One Health interventions.

## Data Availability

The datasets presented in this study can be found in online repositories. The names of the repository/repositories and accession number(s) can be found below: https://www.ncbi.nlm.nih.gov/genbank/, OQ572336–OQ572344, PV646698–PV646710, PV646702–PV646703, and PV646705–PV646709.

## Supplementary material

Supplementary Table 1 Prevalence and distribution of ITS genotypes of *E. bieneusi* in humans in China: summary of published studies.
