# Supplementary material for: Molecular epidemiology of Enterocytozoon bieneusi in humans and domestic animals from Heilongjiang province, China: high animal prevalence, novel genotypes, and possible zoonotic implications in rural households
Source: Front Cell Infect Microbiol. 2026 Aug 24;16:1899714. doi: 10.3389/fcimb.2026.1899714 (PMC13546994; doi:10.3389/fcimb.2026.1899714)
Supplement: Supplementary file 1 [file DataSheet1.pdf]

Supplementary Material 1: Table S1

Prevalence and distribution of ITS genotypes of *E. bieneusi* in humans in China.

Region	Population	Examined no	Positive no	Prevalence (%)	ITS genotype (n)	Reference
Chongqing	Diarrhea children	93	11	11.8	PigEBITS5 (3), CC2 (2), CQ-H1 (2), CQ-H2 (2), CQ-H3 (2), CQ-H4 (1)	[Ding S et al.,2018]
	Diarrhea patients	132	14	10.6	—	[Zang M et al.,2021]
Guangxi	Facility workers	25	6	24.0	D (3), CM1 (2), MEB5 (1)	[Li J et al.,2021]
	Villagers	25	1	4.0	D (1)	
	HIV positive patients	285	33	11.6	D (11), type IV/K (7), PigEBITS7 (7), EbpC (4), GX25 (1), GX456 (1), GX458 (1)	[Liu H et al.,2017]
Henan	HIV-positive participants	683	39	5.7	EbpC (18), D (7), type IV (6), PigEBITS7 (1), Peru8 (1), EbpD (1), Henan-I-V (1each)	[Wang L et al.,2013a]
	HIV-negative participants	683	29	4.2	EbpC (21), D (5), type IV (1), Peru11 (1), Unknown (1)	
	Children patients	2284	27	1.18	D (17), J (2), PigEBITS7 (1), BEB6 (1), CM8 (1)	[Yu F et al.,2019]
Shandong	Diarrhea Patients	44	5	11.4	—	[Zang M et al.,2021]
Shanghai	Diarrhea children	252	34	13.5	—	[Liu H et al.,2014]
	Children patients	573	24	4.2	Peru11 (6), EbpA (2), SH2 (3), SH1 (1), SH3 (1), SH4 (1), EbpC (1), SH5-8 (1 each), D (1), SH9-12 (1 each)	[Wang L et al.,2013b]
Hubei	Diarrhea Patients	20	1	5.0	—	[Zang M et al.,2021]
Heilongjiang	Nondiarrhea and	255	19	7.5	NEC5 (3), EbpC(9), CS-4(3), Henan-IV(2),	[Yang J et al.,2014]

	diarrhea children				NEC1(1), NEC2(1), NEC3(1), NEC4(1)	
	HIV positive participants	384	56	14.6	D (37), EbpC (1), CHN-H1(4), CHN-H2(1), CHN-H3(1), NA (12)	[Zhao W et al.,2022]
	HIV negative participants	199	2	1.0	—	
	Diarrhea patients	222	3	1.0	D (1), YCHH1 (1), YCHH2 (1)	[Zhou K et al.,2022]
	Cancer patients	381	5	1.3	D (4), HLJ-CP1(1)	[Zhang W et al.,2017]
Hubei	Diarrhoea children	500	1	0.2	D (1)	[Wang T et al.,2017]
Jilin	Diarrhea children	40	9	22.5	CHN1 (5), CHN3 (4), CHN4 (3), I (3), J (3), CHN2 (2)	[Zhang X et al.,2011]
Sichuan	Diarrhea patients	124	9	7.3	—	[Qiu L et al.,2017]
Xinjiang	Children	609	36	5.9	A (3), CHN6 (1), CXJH1 (1), CXJH2 (1), CXJH3 (1), D (6), EbpA (3), KB-1 (1), NIA1 (19)	[Qi M et al.,2020]
Yunnan	Villagers	289	24	8.3	Peru 6 (21), YN104 (1), YN241 (1), YN249 (1)	[Gong B et al.,2019]
	College students	426	1	0.2	EbpC (1)	[Yan H et al.,2021]
Total		8528	389	4.6	D (94), EbpC (55), Peru 6 (21), NIA1 (19), type IV(14), NA (12), PigEBITS7 (9), type K (7), EbpA (5), CHN1 (5), CHN3 (4), PigEBITS5 (3), SH2 (3), NEC5(3), CS-4 (3), CHN4 (3), I (3), J (3), A (3), CHN2 (2), CC2(2), CQ-H1 (2), CQ-H2 (2), CQ-H3 (2), Henan-IV (2), CQ-H4(1),CM1 (2), MEB5 (1), GX25 (1), GX456(1), GX458 (1), Peru8(1), EbpD (1), Henan-I-V (1each), Peru11 (7), Unknown (1), SH1 (1), SH3(1), SH4 (1), SH5-8 (1each), SH9-12	

		(1each), NEC1 (1), NEC2 (1), NEC3 (1), NEC4 (1), CHN-H1 (4), CHN-H2 (1), CHN-H3 (1), YCHH1 (1), YCHH2 (1), HLJ-CP1 (1), CHN6 (1), CXJH1 (1), CXJH2 (1), CXJH3 (1), KB-1 (1), YN104 (1), YN241 (1), YN249 (1)

[a]Four genotypes in **Online Resource 1** have been changed to the first published names instead of genotype names described in original papers (SH5 to BEB6, CHN1 to BEB4, SH12 to type IV, SH7 to Henan-I).
