# Supplementary material for: Molecular epidemiology of Enterocytozoon bieneusi in humans and domestic animals from Heilongjiang province, China: high animal prevalence, novel genotypes, and possible zoonotic implications in rural households
Source: Front Cell Infect Microbiol. 2026 Aug 24;16:1899714. doi: 10.3389/fcimb.2026.1899714 (PMC13546994; doi:10.3389/fcimb.2026.1899714)
Supplement: Supplementary file 2 [file DataSheet2.docx]

**Supplementary Material 2: Table S2**

Homology analysis of ITS region of the SSU rRNA genes of *E. bieneusi* representative sequences at the nucleotide level.

| Host | Village (Isolate ID) | Genotype | GenBank ID (Host-Country) | Homology | No.of nucleotide differences | Phylogenetic group (No.) |
| --- | --- | --- | --- | --- | --- | --- |
| Human, Cattle | DFH (H42); SF (H11, H14); QJ (B42, B44) | CHLJ-H1 | KX383644 (Sika deer-China) | 99.72% | 1 | 2 |
| Human | GA (H91) | CHLJ-H2 | KT750165 (Fox- China), PP660645 (Reed Vole-China), LC436478 (Korean water deer-South Korea) | 99.59% | 1 | 1 |
| Human | XF (H74) | CHLJ-H3 | MN758743 (Bullfrog-China) | 99.59% | 1 | 1 |
| Human, Pig | XF (H76); CF (P88) | CHLJ-H4 | OR911594 (Rock Pigeon-Czech Republic), KR815517 (Sika deer -China), MN758752 (Bullfrog-China) | 99.59% | 1 | 1 |
| Human | XL (H116) | CHLJ-H5 | OR911594 (Rock Pigeon-Czech Republic), KR815517 (Sika deer -China) | 99.59% | 1 | 1 |
| Goose | YHA (E32) | CHLJ-E1 | MN136777 (Homo sapiens-China), KP057600 (Pere David's Deer-China), MK478058 (Bamboo rat-China) | 99.59% | 2 | 1 |
| Goose | YHA (E39) | CHLJ-E2 | MN136777 (Homo sapiens-China), KP057600 (Pere David's Deer), MK478058 (Bamboo rat-China) | 99.59% | 1 | 1 |
| Goose | YHA (E55) | CHLJ-E3 | MT773147 (Homo sapiens-China), KX869923 (Dog-China), MK681457 (Wild boar-China) | 99.72% | 1 | 1 |
| Goose | YHA (E70) | GHLJ-E4 | MT773147 (Homo sapiens-China), KX869923 (Dog-China, MK681457 (Wild boar-China) | 99.72% | 1 | 1 |
| Goat | GA (Sh40) | CHLJ-S1 | OM948686(Homo sapiens-China), LC436478 (Korean water deer-South Korea), PP660645 (Reed Vole-China) MK168302 (Blue peafowl -China) | 99.72% | 1 | 1 |
| Chicken | YHA (C209) | CHLJ-C1 | MT773147 (Homo sapiens-China), KX869923 (Dog-China), MK681457 (Wild boar-China) | 99.72% | 1 | 1 |
| Chicken | YHA (C221) | CHLJ-C2 | PP158567 (Rattus tanezumi-China), PV364864 (Pig-Bangladesh), PP158568 (Rattus norvegicus-China) | 99.72% | 1 | 1 |
| Chicken | QJ (C174) | CHLJ-C3 | OR911594 (Rock Pigeon-Czech Republic), KR815517 (Sika deer-China) | 99.59% | 1 | 1 |
| Chicken | CF (C246) | CHLJ-C4 | OR911594 (Rock Pigeon-Czech Republic), KR815517 (Sika deer-China), MN758752 (Bullfrog-China) | 99.59% | 1 | 1 |
| Pig | CF (P102, P110); LL (P161) | CHLJ-P1 | KF607052 (Pig-China), MG602795 (Bactrian camel-China), MK778892 (Pig-China), KU245701 (Dairy cattle-China) | 99.59% | 1 | 1 |
| Pig | XL (P125) | CHLJ-P2 | MZ172787 (Homo sapiens-Thailand), OQ469426 (Flies-China), OQ520125 (Flies-China) | 99.59% | 1 | 1 |
| Pig | XL (P135) | CHLJ-P3 | OQ520126 (Wild boar-China), KX670578 (Wild boar-China), MK778894 (Pig-China) | 99.59% | 1 | 1 |
| Pig | XL (P149) | CHLJ-P4 | OR911594 (Rock Pigeon-Czech Republic), KR815517 (Sika deer -China), MK681457(Wild boar-China), MT193871 (Cattle-China) | 99.18% | 2 | 1 |
| Pig | CF (P84) | CHLJ-P5 | OR911594 (Rock Pigeon-Czech Republic), KR815517 (Sika deer -China), MN758752 (Bullfrog-China) | 99.59% | 1 | 1 |
| Sheep | GA (Sh19, Sh27, Sh30, Sh32, Sh35) | CHS7 | OL604155 (Sheep-China:Shanxi), KP262392 (Sheep-China) | 100% | 0 | 2 |
| Goat | SF (S13, S14) | COS-II | MH822620 (Goat-China), PP453723 (Sheep-China) | 100% | 0 | 2 |
| Sheep | GA (Sh15, Sh16, Sh17, Sh18, Sh20, Sh21, Sh22, Sh23, Sh24, Sh25, Sh28, Sh31, Sh33, Sh34, Sh36, Sh37, Sh38, Sh39, Sh41, Sh42, Sh43, Sh44); YHA (Sh45, Sh59) | BEB6 | KX383615 (Sika deer-China: Henan), MT674905 (Sheep-China) | 100% | 0 | 2 |
| Chicken | YH (C8) | CHN-H1 | MG255733 (Homo sapiens-China) | 100% | 0 | 1 |
| Human, Chicken, Pig, Dog, Cattle, Goose, Sheep | SF (C96); DFH (P11), YHA (C191, C214, C225, C229); QJ (B22); XL (D19); (P128, P129, P148, P153, P156, P155, P157, P159); CF (H111, H112); QJ (H65, H68); (C162, C173); CF (C237, C238, C239, C242, C243, C244, C245, C247, C248, C249, C250); YHA (E46); CF (E72, E73, E77, E78, E79, E80, E81); XL (E82, E83); CF (P73, P74, P75, P76, P77, P78, P79, P80, P81, P82, P83, P85, P90, P91, P94, P89, P93, P108, P111); LL (P162) | EbpC | MG581432 (Tibetan pig-China), MN179305 (Cygnus cygnus-China), OQ520132 (Pig-China), KR815517 (Sika deer-China), MG581432 (Tibetan pig-China), MN179305 (Cygnus-China), OQ520132(Pig-China) | 100% | 0 | 1 |
| Human, Pig, Chicken, Fox, Goose | SF (P42, P43, P45, P46, P48, P51, P54, P55, P57, P58, P59); GA (C143); QJ (H59); YHA (P66, P67, P69); YHA (F22); YHA (C194, C200); YHA (E27) | D | MT652647 (Giraffe-China), MK932867 (Cat-Spain) | 100% | 0 | 1 |
| Human, Goose, Pig, Chicken, Cattle | YHA (E31, E52); DFH (P12); YH (P30, P31); YHA (C181, C183, C201, C203); CF (P97, P98, P99, P103, P104, P106, P107, P109, P118); XL (P142, P144, P145, P160); QJ (H99); LL (P164) | EbpA | OQ520128 (Flies-China), MK778907 (Pig-CHina), KJ668725 (Dog-China), MG581432 (Tibetan pig-China), MN179305 (Cygnus-China), OQ520132 (Pig-China) | 100% | 0 | 1 |
| Pig, Chicken | CF (P115, P113, P114, P116, P120); CF (C240) | H | AF135835 (Pig-Germany), KP318000 (Pig-Brazil), KT922240 (Calf-Ethiopia), MT231505 (Calf-Ethiopia) | 100% | 0 | 1 |
| Pig | XL (P126, P131, P87) | PigEBITS5 | MK681477 (Wild boar-China), KJ728799 (Pig-tailed macaque-China), MK681476 (Wild boar-China) | 100% | 0 | 1 |
| Pig | XL (P151) | Henan-III | JF691566 (Homo sapiens-China), MN758756 (Bullfrog-China) | 100% | 0 | 1 |
